# Expression pattern of class I histone deacetylases in vulvar intraepithelial neoplasia and vulvar cancer: a tissue microarray study

**DOI:** 10.1186/1471-2407-11-463

**Published:** 2011-10-26

**Authors:** Nicolas Samartzis, Patrick Imesch, Konstantin J Dedes, Eleftherios P Samartzis, André Fedier, Daniel Fink, Rosmarie Caduff, Mathias K Fehr

**Affiliations:** 1 Department of Gynecology, University Hospital Zurich, Frauenklinikstrasse 10, 8091 Zurich, Switzerland; 2 Department of Pathology, University Hospital Zurich, Schmelzbergstrasse 12, 8091 Zurich, Switzerland; 3 Department of Gynecology and Obstetrics, Hospital of Frauenfeld, 8051 Frauenfeld, Switzerland

**Keywords:** Histone deacetylase, epigenetics, vulvar intraepithelial neoplasia, vulvar squamous cell cancer, tissue microarray, immunohistochemistry

## Abstract

**Background:**

Epigenetic regulation is an important mechanism leading to cancer initiation and promotion. Histone acetylation by histone deacetylases (HDACs) represents an important part of it. The development of HDAC inhibitors has identified the utility of HDACs as a therapeutic target. Little is known about the epigenetic regulation of vulvar intraepithelial neoplasia (VIN) and vulvar squamous cell cancer (VSCC). In this study, the expression of class I HDACs (HDAC 1, 2 and 3) was compared in a series of VIN and VSCC tissues.

**Methods:**

A tissue micro array (TMA) with specimens from 106 patients with high-grade VIN and 59 patients with vulvar cancer was constructed. The expression of HDACs 1, 2 and 3 were analyzed with immunohistochemistry (IHC). The nuclear expression pattern was evaluated in terms of intensity and percentage of stained nuclei and was compared between vulvar preinvasive lesions and vulvar cancer.

**Results:**

HDAC 2 expression was significantly higher in VIN than in VSCC (p < 0.001, Fisher's test). Also, 88.7% (n = 94/106) of VIN samples and only 54.5% (n = 31/57) of VSCC samples were scored at the maximum level. Conversely, HDAC 3 expression was significantly higher in VSCC (93%, 53/57) compared to VIN (73.6%, 78/106, p = 0.003), whereas only a small difference in the expression of HDAC 1 was found between these two entities of vulvar neoplasia.

**Conclusions:**

These results suggest that epigenetic regulation plays a considerable role in the transformation of VIN to invasive vulvar neoplasia.

## Background

Invasive vulvar squamous cell carcinomas (VSCC) represent the fourth most common type of malignant tumor of the female genital tract in the United States, with an estimated 3, 580 new cases and 900 deaths in 2009 [1]. Recently, a significant increase of precancerous lesions and invasive vulvar carcinomas has been observed in industrialized countries. The incidence of invasive and vulvar intraepithelial neoplasia (VIN) has risen 2.4% per year in the U.S. from 1992 to 1998 [2]. A Scandinavian study describes an increase in VIN incidence more than 4 times from 1973 to 2000 and of 20% for invasive vulvar cancer [3]. The described increase in incidence is seen primarily in younger women, whereas in elderly women, the incidence rates of vulvar cancer have remained relatively stable over the past few decades.

VIN is most commonly treated by local excision, laser evaporation, or a combination of both methods, to preserve vulvar function and morphology. The preferred treatment modality for VSCC is surgery whenever feasible. Small tumors are treated by wide local excision, with or without partial or radical vulvectomy, combined with lymph node staging via sentinel lymph node biopsy or inguinofemoral lymphadenectomy if lymph node metastases are present. For patients with extensively involved inguinofemoral lymph nodes, radiotherapy of the pelvis is advantageous. For patients with recurrent or metastatic disease, irradiation and chemotherapy offer some benefit; however, response rates are regarded as low [4,5]. Targeted therapies for VSCC have not yet been established in clinical practice [6], but given the low benefit of conventional chemotherapies, novel systemic treatment modalities are urgently needed for these patients.

Epigenetics characterize the hereditary changes in the pattern of gene expression that are not due to changes in DNA sequence. Genetics and epigenetics interact at all stages of cancer development. Epigenetic alterations in mammalian genomes fall into two main categories: DNA methylation and histone modification. Histones are strongly alkaline proteins that are able to package the DNA and condense it into structural units called nucleosomes. Acetylation and deacetylation of histones are performed by histone acetyltransferase (HATs) and histone deacetylases (HDACs), respectively. HDACs increase the affinity of histone complexes to DNA. The chromatin is thereby more condensed and transcriptionally repressed [7-9].

Additionally HDACs can modify proteins other than histones, such as transcription factors (e.g. p53, E2F, pRb). Acetylation can also affect protein stability and protein-protein interactions. Therefore, HDACs are emerging as important regulators of cell growth, differentiation, and apoptosis [10,11]. There are at least eighteen deacetylase enzymes known in human cells, categorized into four classes: class I (HDAC 1, 2, 3, 8), class II (HDAC 4, 5, 6, 7, 9, 10), class III (SIRT 1-7) and class IV (HDAC 11). HDAC1 and HDAC2 are one of the best-characterized HDACs. However, the isoenzyme-specific biological functions of HDACs are still mostly unknown [8]. It has been postulated that dysregulated function of HDACs leads to cancer formation and development [11]. Altered HDAC expression is observed in a variety of cancer types, such as prostate adenocarcinoma [12], gastric carcinoma [13], colorectal carcinoma [14], cervical dysplasia and endometrial stromal sarcoma [15]. In vulvar intraepithelial neoplasia and vulvar cancer, no data on HDAC expression has been published.

The aim of this study was to analyze the expression of the class I HDACs 1, 2 and 3 by immunohistochemistry in a series of VIN and VSCC samples using the tissue microarray technique and to correlate the finding with the clinicopathological features of the patients.

## Methods

### Patient characteristics

One-hundred-six patients diagnosed with high-grade VIN and 59 patients with VSCC between 1993 and 2006 at the Institute of Pathology, University Hospital Zurich were included in this study. The study was approved by the local ethics committee (ref. number StV 08/2006). Histological diagnosis was established according to the guidelines of the International Society for the Study of Vulvovaginal Disease (ISSVD) [16].

Clinical data were available for 158 of the 165 cases. Follow-up data of at least six months were available for 74 of the 106 patients with VIN and 25 of the 59 patients with VSCC. Mean follow-up time was 67.8 months (SD ± 41.8, range 8 - 169 months) and 50.6 months (SD ± 42.7, range 6 - 149 months) in patients with VIN and VSCCs, respectively. Table 1 shows the patient's age and p16 status in VIN and VSCC. Table 2 shows the clinicopathological data of the patients with VSCC included in the study.

**Table 1:** Patients' age and p16 status in different diagnostic groups

	p16	n	age mean	SD	range
**VIN**	pos	93	49.1	± 14.6	22 - 89
	Neg	13	74.3	± 14.9	35 - 93
**VSCC**	Pos	31	61.8	± 14.3	37 - 90
	Neg	26	77.3	± 10.3	53 - 93

Mean age, standard deviation (SD) and range are given in years.

**Table 2:** Clinicopathological features of patients with VSCC

	n	percentage
**T stage**		
1	22	37.3%
2	24	40.7%
3	9	15.3%
4	0	0.0%
Not available	4	6.8%
**Lymphe node metasases**		
None	24	40.7%
Unilateral	14	23.7%
Bilateral	6	10.2%
Not available	15	25.4%
**Distant metastases**		
No	28	47.5%
Yes	1	1.7%
Not available	30	50.8%
**FIGO**		
I	16	27.1%
II	12	20.3%
III	13	22.1%
IV	5	9.5%
Not available	13	22.0%
**Tumor differentiation**		
Well	13	22.0%
Moderate	30	50.8%
Poor	16	27.1%

### Tissue Microarray construction

Two tissue microarrays (TMA), one for the VIN and one for the VSCC cases, were constructed using a semiautomatic tissue arrayer (Beecher Instruments, Woodland, USA) as previously described [17]. Areas involving vulvar cancer or VIN were marked on hematoxylin/eosin-stained sections. Cylindrical cores 0.6 mm in diameter were punched out of the corresponding paraffin embedded block and inserted into a recipient block. Two different spots from each tumor were punched out.

### Immunohistochemistry

TMA sections (2.5 μm) were transferred to glass slides, followed by immunohistochemical analysis according to the Ventana automat protocols. The following antibodies were used: polyclonal rabbit IgG antibody against HDAC 1 (dilution 1:2, Abcam Limited, clone: ab15316, UK-CB4 0FL Cambridge, United Kingdom), monoclonal mouse IgG antibody against HDAC 2 (dilution 1:1000, Abcam Limited, clone: ab12169, UK-CB4 0FL Cambridge, United Kingdom) and monoclonal mouse IgG antibody against HDAC 3 (dilution 1:500, Becton Dickinson, clone: 611125, NJ-07417 Franklin Lakes, United States of America).

Additionally, immunohistochemical staining with p16 (dilution 1:200, Santa Cruz Biotechnology, clone: sc-56330, CA-95060 Santa Cruz, United States of America) and Ki-67 (dilution 1:20, DAKO, clone: M7240, DK-2600 Glostrup, Denmark) was performed.

P16, a tumor suppressor gene, inhibits cyclin dependent kinase 4 (CDK4), 6 (CDK6) and retinoblastoma protein (pRb) and subsequently blocks the passage from G1 into S phase [18]. Human papilloma virus (HPV) inactivates p53 and pRb with its E6 and E7 oncogenic proteins after infection of epithelial cells, which results in an overexpression of p16 versus a negative feedback control of pRb [19,20]. Therefore, p16 expression has been established as a surrogate marker for HPV infection and is used for pathomorphological investigation [21-23]. Ki-67 (Mib-1) is expressed during cellular proliferation and, therefore, is used as a marker to determine the growth fraction in tissue samples (proliferation index) [24].

Immunohistochemical staining of HDAC isoforms was scored by applying a semiquantitative immunoreactivity scoring system (IRS). Therefore the percentage of positive cells was categorized as none (0), less then 10% of the cells (1), 10-50% of the cells (2), 51-80% of the cells (3), and more then 80% of the cells (4). The intensity was graded as absent (0), weakly positive (1+), moderately positive (2+) or strongly positive (3+). The IRS score results from multiplying the area-score with the intensity of immunoreactivity, as described elsewhere [25]. It ranges from 0 to 12. Nuclear staining of HDACs was considered positive, whereas cytoplasmic staining was regarded as nonspecific. Both TMAs were scored by two observers (N.S. & R.C.) who were blinded to the clinicopathological information of each sample. The two cores of each individual tumor were scored separately, and the mean score of the two twin tissue cores was attributed to a single patient.

To assess correlations and associations between expression of HDACs and clinicopathological parameters, Spearman's rho (bivariate correlation analysis), Fisher's exact test and χ-square tests were applied, where appropriate. p-values of < 0.05 were considered significant. SPSS 18.0 package software (SPSS Inc., Chicago/Illinois, USA) was used.

## Results

### HDAC expression in VIN and VSCC

Nuclear HDAC 1, 2 and 3 staining could be evaluated in 163 of 165 cases (98.8%). In 9 cases out of 163 (5.5%), only one of both tissue cores could be analyzed. In the non-evaluable cases, the cores lacked sufficient epithelial cells.

In VIN, mean IRS-scores of HDAC 1, HDAC 2 and HDAC 3 were 9.99, 11.56 and 10.88, and they were 9.83, 9.75 and 11.72 in VSCC, respectively. The median IRS-score was 12 for every HDAC isoform in both VIN as well as VSCC. Hence, the cut-off point for statistical analysis was taken at a score level of 12. Results were, therefore, dichotomized into a "HDAC high" group (IRS = 12) and a "HDAC low" group (IRS < 12).

**Figure 1:**
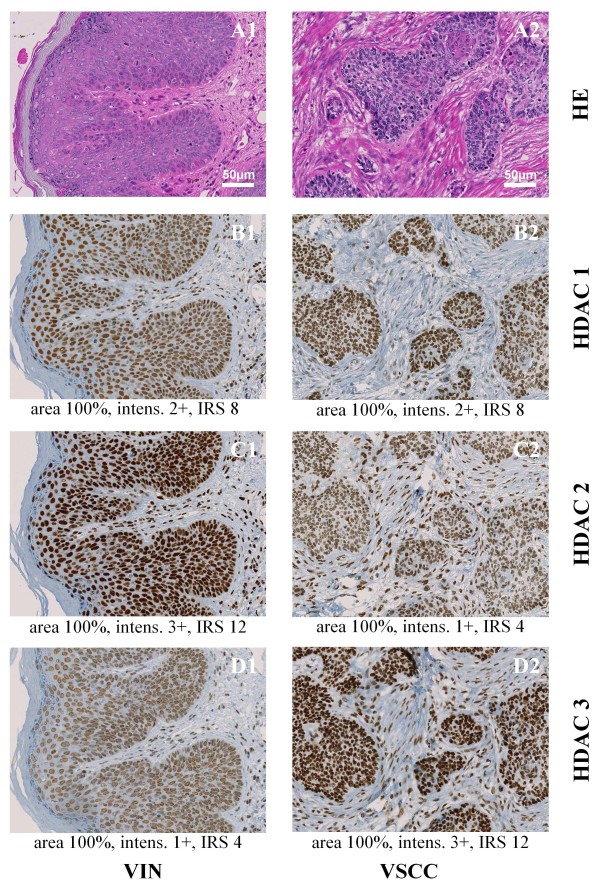
**Representative tissue samples of one VIN microarray core (A1, B1, C1, D1) and one VSCC microarray core (A2, B2, C2, D2) stained with hematoxylin/eosin (HE) (A1 and A2) and nuclear immunohistochemical reaction with class I HDAC antibodies: HDAC 1 (B1 and B2), HDAC 2 (C1 and C2) and HDAC 3 (D1 and D2); Magnification × 200**. Under each immunohistochemical stain, the percentage of positive epithelial cells within the tissue core (area), the intensity of immunoreactions (intens.) and the immunoreactivity score (IRS) are indicated.

**Figure 2:**
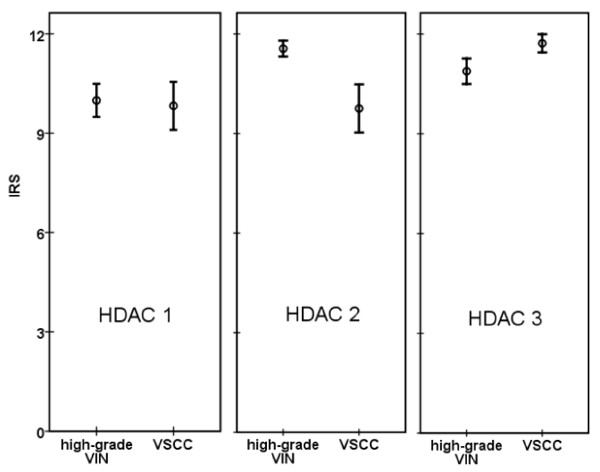
**HDACs immunoreactivity score (IRS) in VIN and vulvar cancer tissue**. The mean IRS is represented by a circle, and the 95% confidence interval is represented by an error bar. The confidence intervals of VIN and VSCC in HDAC 2 and HDAC 3 do not overlap in contrast to that of HDAC 1.

The Association of HDAC 1, 2 and 3 expressions with clinicopathological parameters is summarized in Table 3. HDAC 2 expression was significantly higher in VIN than in VSCC (p < 0.001, Fisher's test). 88.7% (n = 94/106) of VIN samples were scored at the maximum level (IRS 12); vulvar cancer samples only in 54.5% (n = 31/57). Conversely, HDAC 3 expression was significantly higher in VSCC (93%, 53/57) compared to VIN (73.6%, 78/106), and p = 0.003. No significant distribution was found for HDAC 1 within vulvar neoplasias. In fact, the percentage of tissue samples with high HDAC 1 protein expression is almost equal between VIN (57.5%) and VSCC (56.1%). The reciprocal expression pattern of HDAC 2 and 3 is illustrated in representative tissue examples in Figure 1 and graphically visualized in Figure 2.

**Table 3:** Association of HDACs with clinicopathological parameters

	Total	HDAC1 low	HDAC1 high	p	HDAC2 low	HDAC2 high	p	HDAC3 low	HDAC3 high	p
**All Cases**	163	70 (42.9%)	93 (57.1%)	--	38 (23.3%)	125 (76.7%)	--	32 (19.6%)	131 (80.4%)	--
**VIN**	106	45 (42.5%)	61 (57.5%)	0.87	12 (11.3%)	94 (88.7%)	**< 0.001**	28 (26.4%)	78 (73.6%)	**0.003**
**VSCC**	57	25 (43.9%	32 (56.1%)		26 (45.6%)	31 (54.4%)		4 (7.0%)	53 (93.0%)	
**p16 neg**	39	17 (43.6%)	22 (56.4%)	1.00	10 (25.6%)	29 (74.4%)	0.67	4 (10.3%)	35 (89.7%)	0.109
**p16 pos**	124	53 (42.7%)	71 (57.3%)		28 (22.6%)	96 (77.4%)		28 (22.6%)	96 (77.4%)	
**Age ≤ 60**	93	41 (44.1%)	52 (55.9%)	0.752	22 (23.7%)	71 (76.3%)	1.00	21 (22.6%)	72 (77.4%)	0.322
**Age > 60**	70	29 (41.4%)	41 (58.6%)		16 (22.9%)	54 (77.1%)		11 (15.7%)	59 (84.3%)	

**VSCC°**										
**pT1**	20	7 (35.0%)	13 (65.0%)	0.556*	5 (25.0%)	15 (75.0%)	0.057*	1 (5.0%)	19 (95.0%)	0.831*
**pT2**	24	11 (45.8%)	13 (54.2%)		13 (54.2%)	11 (45.8%)		2 (8.3%)	22 (91.7%)	
**pT3**	9	5 (55.6%)	4 (44.4%)		6 (66.7%)	3 (33.3%)		1 (11.1%)	8 (88.9%)	
**pN0**	23	9 (39.1%)	14 (60.9%)	0.763	9 (39.1%)	14 (60.9%)	0.366	1 (4.3%)	22 (95.7%)	1.00
**pN1/2**	20	9 (45.0%)	11 (55.0%)		11 (55.0%)	9 (45.0%)		1 (5.0%)	19 (95.0%)	
**G1**	12	5 (41.7%)	7 (58.3%)	0.009*	7 (58.3%)	5 (41.7%)	0.602*	1 (8.3%)	11 (91.7%)	0.513*
**G2**	29	8 (27.6%)	21 (72.4%)		12 (41.4%)	17 (58.6%)		1 (3.4%)	28 (96.6%)	
**G3**	16	12 (75%)	4 (25.0%)		7 (43.8%)	9 (56.3%)		2 (12.5%)	14 (87.5%)	

**VIN**										
**Ki-67 ≤ 10%**	11	9 (81%)	2 (18.2%)	**0.008**	3 (27.3%)	8 (72.7%)	0.109	4 (36.4%)	7 (63.6%)	0.476
**Ki-67 > 10%**	95	36 (37.9%)	59 (62.1%)		9 (9.5%)	86 (90.5%)		24 (25.3%)	71 (74.7%)	
**VSCC**										
**Ki-67 ≤ 10%**	19	8 (42.1%)	11 (57.9%)	1.00	14 (73.7%)	5 (26.3%)	**0.004**	2 (10.5%)	17 (89.5%)	0.594
**Ki-67 > 10%**	38	17 (44.7%)	21 (55.3%)		12 (31.6%)	26 (68.4%)		2 (5.3%)	36 (94.7%)	

HDAC immunoreactivity score (IRS) has been dichotomized in two groups; HDAC 1 to 3 "high" represent tissue samples with a IRS of 12, HDAC 1 to 3 "low", a IRS less than 12. Clinicopathological parameters investigated in this study are listed in the first column; Vulvar intraepithelial neoplasia (VIN), vulvar squamous cell cancer (VSCC); p-16 as a surrogate marker for human papilloma virus (HPV) infection as described before [21-23]; patient younger than 60 years (Age ≤ 60), patient equal or elder than 60 years (Age > 60); related pTNM-stage and tissue differentiation (G1 to G3) in VSCC. Nuclear Ki-67 protein is a marker for cell proliferation. "Ki-67 ≤ 10%" means that 10 percent or less of the cells are proliferating.

Percentages in parentheses are according to the total number of samples in the first column. P-values result from the association of HDAC low and high groups to one parameter, such as tissue type VIN versus VSCC. Where not specified, Fisher's exact test was used to calculate p-values. P-values labeled by * have been obtained by χ-square test. For a facilitated reading, corresponding data to p-values are in one grid each.

° T-stage: 6 cases missing at all, no cases of pT4; N-stage: 15 cases missing; M-stage: not shown, 30 cases with no investigation and or data missing.

In a bivariate correlation analysis, IRS scores of the three HDAC isoforms correlated significantly with each other in VIN samples, whereas no correlation between isoform 2 and 3 was found in VSCC.

### Correlation of HDAC expression with clinicopathological parameters

There was a moderate correlation between HDAC 2 and pT stage in VSCC (p = 0.009), whereas no significant association between tumor size and high HDAC 2 expression was observed. A high proliferation index (Ki-67 area) correlated with high HDAC 1 (p = 0.008, cc = 0.21) and HDAC 2 (p < 0.001, cc = 0.36) expression. Using cut-off levels of 10%, this correlation was confirmed (Table 3). Similar to this findings, applying a cut-off level of 25% results in a significant associations between Ki-67 and HDAC 1 in VIN (p = 0.012) and Ki-67 and HDAC 2 in the VSCC group (p = 0.035).

In the grouped analyses, no significant association between p16 positivity and patient age with HDAC expression was observed (Table 3). In fact, almost equal frequencies were found between p16 and HDAC 1 as well as between patient age and HDAC 2. There was no significant association between tumor size (pT) and high HDAC 2 expression.

## Discussion

This study shows that class I HDACs are highly expressed in the majority of VIN and VSCC; however, the expression patterns differ between VIN and VSCC. High HDAC-2 protein expression is found more often in VIN, and high HDAC-3 protein expression is found more often in VSCC. These two types of HDACs are neither associated with patient age nor with level of p16 expression. Therefore, the observed differences are not explained by the difference of the average age or frequency of p16 positivity in VIN and VSCC. The immunohistochemical staining showed high intensity in the majority of tissue samples, and negative results were not found. HDAC 3 was the most intensely expressed isoform of all three class I HDACs.

Based on the expression pattern of histone deacetylating proteins, we hypothesized that epigenetic regulation plays a major role in the pathogenesis of invasive vulvar cancer, as has been demonstrated for several other malignancies [12-15]. The transformation of non-invasive to invasive vulvar neoplasia may be promoted by epigenetic regulation. To our knowledge, this report is the first on class I HDAC expression in vulvar cancer or vulvar intraepithelial neoplasia.

P16 has been proposed as a surrogate marker for HPV associated neoplasia [21-23]. We found no difference between class I HDAC expression in p16-negative or p16-positive tumor tissue. Therefore, the regulation of gene expression by HDACs seems to be independent of HPV infection.

One mechanism by which HDACs appear to stimulate tumor cell growth is through the repression of the tumor suppressor gene, p21 [26]. In this study, high expression of HDAC 1 in VIN and high expression of HDAC 2 in VSCC were associated with increased cell proliferation, as defined by Ki-67. This result supports previous findings in other tumor types, such as prostate cancer and colorectal cancer [27,28]. With the exception of the significant correlation of HADC 1 and 2 with the proliferation marker Ki-67, no relation was found between HDAC expression and clinicopathological features. Still, the utility of these results must be considered carefully because of the lack of a full clinical data set.

The epigenetic regulation of HDACs has recently been explored as a therapeutic target by the discovery and development of HDAC-inhibitors (HDACi). *In-vitro *data suggest that HDACi induce cell cycle arrest, differentiation, and apoptosis [15,29-32]. The antitumor effects of HDACi emphasize the important role of HDACs in cancer development. However, HDACi affects the activity of several enzymes, and it is difficult to identify the particular functions of different HDAC isoforms involved in cancerogenesis. Several HDACi are currently under clinical investigation involving various hematological malignancies and solid tumors, of which vorinostat has already been approved for the systemic treatment of cutaneous T cell lymphoma [33]. Particularly, in oral squamous cell cancer, there are different phase I and II trials using HDAC-i as a monotherapy or in combination with other agents. This treatment method is of interest regarding VSCC [34-37].

## Conclusions

In summary, HDACs, the targets of HDACi, are highly expressed in the majority of VIN and VSCC and show a different expression pattern among these two tissue types irrespective of the HPV-related etiology. Investigations of HDAC-i for the topical or systemic treatment of VIN and VSCC are warranted.

## Competing interests

The authors declare that they have no competing interests.

## Authors' contributions

NS constructed the tissue microarray (TMA), scored the immunohistochemical (IHC) staining and drafted the manuscript. PI prepared the study design, interpreted data and drafted part of the manuscript. KJD evaluated the patients for study inclusion and contributed to the conclusion section of the manuscript. EPS performed statistical analyses and drafted part of the result section. AF contributed to the interpretation of data and developed figures for the manuscript. DF assisted with the interpretation of data and contributed to the draft of the manuscript. RC revised diagnosis of the paraffin embedded tissue samples and analyzed the IHC-staining of the two TMAs. MFK conceived the study, collected data and coordinated the procedures during this study. All authors read and approved the final manuscript.

## Pre-publication history

The pre-publication history for this paper can be accessed here:

http://www.biomedcentral.com/1471-2407/11/463/prepub

## References

[B1] Jemal A Siegel R Ward E Hao Y Xu J Thun MJ Cancer statistics, 2009 CA Cancer J Clin 2009 59 4 225 249 10.3322/caac.20006 19474385

[B2] Howe HL Wingo PA Thun MJ Ries LA Rosenberg HM Feigal EG Edwards BK Annual report to the nation on the status of cancer (1973 through 1998), featuring cancers with recent increasing trends J Natl Cancer Inst 2001 93 11 824 842 10.1093/jnci/93.11.824 11390532

[B3] Judson PL Habermann EB Baxter NN Durham SB Virnig BA Trends in the incidence of invasive and in situ vulvar carcinoma Obstet Gynecol 2006 107 5 1018 1022 10.1097/01.AOG.0000210268.57527.a1 16648405

[B4] Moore DH Chemotherapy and radiation therapy in the treatment of squamous cell carcinoma of the vulva: Are two therapies better than one? Gynecol Oncol 2009 113 3 379 383 10.1016/j.ygyno.2009.01.004 19232700

[B5] Ghurani GB Penalver MA An update on vulvar cancer Am J Obstet Gynecol 2001 185 2 294 299 10.1067/mob.2001.117401 11518882

[B6] Manchana T Ittiwut C Mutirangura A Kavanagh JJ Targeted therapies for rare gynaecological cancers Lancet Oncol 11 7 685 693 10.1016/S1470-2045(09)70368-7 20362508

[B7] Bernstein BE Meissner A Lander ES The mammalian epigenome Cell 2007 128 4 669 681 10.1016/j.cell.2007.01.033 17320505

[B8] Jones PA Baylin SB The epigenomics of cancer Cell 2007 128 4 683 692 10.1016/j.cell.2007.01.029 17320506 PMC3894624

[B9] Kouzarides T Chromatin modifications and their function Cell 2007 128 4 693 705 10.1016/j.cell.2007.02.005 17320507

[B10] Glozak MA Rogers MB Retinoic acid- and bone morphogenetic protein 4-induced apoptosis in P19 embryonal carcinoma cells requires p27 Exp Cell Res 2001 268 2 128 138 10.1006/excr.2001.5281 11478839

[B11] Glozak MA Seto E Histone deacetylases and cancer Oncogene 2007 26 37 5420 5432 10.1038/sj.onc.1210610 17694083

[B12] Halkidou K Gaughan L Cook S Leung HY Neal DE Robson CN Upregulation and nuclear recruitment of HDAC1 in hormone refractory prostate cancer Prostate 2004 59 2 177 189 10.1002/pros.20022 15042618

[B13] Song J Noh JH Lee JH Eun JW Ahn YM Kim SY Lee SH Park WS Yoo NJ Lee JY Increased expression of histone deacetylase 2 is found in human gastric cancer APMIS 2005 113 4 264 268 10.1111/j.1600-0463.2005.apm_04.x 15865607

[B14] Huang BH Laban M Leung CH Lee L Lee CK Salto-Tellez M Raju GC Hooi SC Inhibition of histone deacetylase 2 increases apoptosis and p21Cip1/WAF1 expression, independent of histone deacetylase 1 Cell Death Differ 2005 12 4 395 404 10.1038/sj.cdd.4401567 15665816

[B15] Hrzenjak A Moinfar F Kremser ML Strohmeier B Staber PB Zatloukal K Denk H Valproate inhibition of histone deacetylase 2 affects differentiation and decreases proliferation of endometrial stromal sarcoma cells Mol Cancer Ther 2006 5 9 2203 2210 10.1158/1535-7163.MCT-05-0480 16985053

[B16] Sideri M Jones RW Wilkinson EJ Preti M Heller DS Scurry J Haefner H Neill S Squamous vulvar intraepithelial neoplasia: 2004 modified terminology, ISSVD Vulvar Oncology Subcommittee J Reprod Med 2005 50 11 807 810 16419625

[B17] Kononen J Bubendorf L Kallioniemi A Barlund M Schraml P Leighton S Torhorst J Mihatsch MJ Sauter G Kallioniemi OP Tissue microarrays for high-throughput molecular profiling of tumor specimens Nat Med 1998 4 7 844 847 10.1038/nm0798-844 9662379

[B18] Sano T Oyama T Kashiwabara K Fukuda T Nakajima T Immunohistochemical overexpression of p16 protein associated with intact retinoblastoma protein expression in cervical cancer and cervical intraepithelial neoplasia Pathol Int 1998 48 8 580 585 10.1111/j.1440-1827.1998.tb03954.x 9736404

[B19] Santos M Montagut C Mellado B Garcia A Ramon y Cajal S Cardesa A Puig-Tintore LM Ordi J Immunohistochemical staining for p16 and p53 in premalignant and malignant epithelial lesions of the vulva Int J Gynecol Pathol 2004 23 3 206 214 10.1097/01.pgp.0000130108.03231.89 15213596

[B20] Riethdorf S Neffen EF Cviko A Loning T Crum CP Riethdorf L p16INK4A expression as biomarker for HPV 16-related vulvar neoplasias Hum Pathol 2004 35 12 1477 1483 10.1016/j.humpath.2004.09.004 15619206

[B21] Santos M Landolfi S Olivella A Lloveras B Klaustermeier J Suarez H Alos L Puig-Tintore LM Campo E Ordi J p16 overexpression identifies HPV-positive vulvar squamous cell carcinomas Am J Surg Pathol 2006 30 11 1347 1356 10.1097/01.pas.0000213251.82940.bf 17063073

[B22] Biedermann K Dandachi N Trattner M Vogl G Doppelmayr H More E Staudach A Dietze O Hauser-Kronberger C Comparison of real-time PCR signal-amplified in situ hybridization and conventional PCR for detection and quantification of human papillomavirus in archival cervical cancer tissue J Clin Microbiol 2004 42 8 3758 3765 10.1128/JCM.42.8.3758-3765.2004 15297527 PMC497646

[B23] O'Neill CJ McCluggage WG p16 expression in the female genital tract and its value in diagnosis Adv Anat Pathol 2006 13 1 8 15 10.1097/01.pap.0000201828.92719.f3 16462152

[B24] Scholzen T Gerdes J The Ki-67 protein: from the known and the unknown J Cell Physiol 2000 182 3 311 322 10.1002/(SICI)1097-4652(200003)182:3<311::AID-JCP1>3.0.CO;2-9 10653597

[B25] Weichert W Roske A Gekeler V Beckers T Ebert MP Pross M Dietel M Denkert C Rocken C Association of patterns of class I histone deacetylase expression with patient prognosis in gastric cancer: a retrospective analysis Lancet Oncol 2008 9 2 139 148 10.1016/S1470-2045(08)70004-4 18207460

[B26] Marks P Rifkind RA Richon VM Breslow R Miller T Kelly WK Histone deacetylases and cancer: causes and therapies Nat Rev Cancer 2001 1 3 194 202 10.1038/35106079 11902574

[B27] Weichert W Roske A Gekeler V Beckers T Stephan C Jung K Fritzsche FR Niesporek S Denkert C Dietel M Histone deacetylases 1, 2 and 3 are highly expressed in prostate cancer and HDAC2 expression is associated with shorter PSA relapse time after radical prostatectomy Br J Cancer 2008 98 3 604 610 10.1038/sj.bjc.6604199 18212746 PMC2243142

[B28] Weichert W Roske A Niesporek S Noske A Buckendahl AC Dietel M Gekeler V Boehm M Beckers T Denkert C Class I histone deacetylase expression has independent prognostic impact in human colorectal cancer: specific role of class I histone deacetylases in vitro and in vivo Clin Cancer Res 2008 14 6 1669 1677 10.1158/1078-0432.CCR-07-0990 18347167

[B29] Munster PN Troso-Sandoval T Rosen N Rifkind R Marks PA Richon VM The histone deacetylase inhibitor suberoylanilide hydroxamic acid induces differentiation of human breast cancer cells Cancer Res 2001 61 23 8492 8497 11731433

[B30] Imesch P Fink D Fedier A Romidepsin reduces histone deacetylase activity, induces acetylation of histones, inhibits proliferation, and activates apoptosis in immortalized epithelial endometriotic cells Fertil Steril 2010 10.1016/j.fertnstert.2010.04.052 20605144

[B31] Li X Chen BD Histone Deacetylase Inhibitor M344 Inhibits Cell Proliferation and Induces Apoptosis in Human THP-1 Leukemia Cells Am J Biomed Sci 2009 1 4 352 363 20526416 10.5099/aj090400352 PMC2880493

[B32] Hrzenjak A Moinfar F Kremser ML Strohmeier B Petru E Zatloukal K Denk H Histone deacetylase inhibitor vorinostat suppresses the growth of uterine sarcomas in vitro and in vivo Mol Cancer 2010 9 49 10.1186/1476-4598-9-49 20202195 PMC2843655

[B33] Tan J Cang S Ma Y Petrillo RL Liu D Novel histone deacetylase inhibitors in clinical trials as anti-cancer agents J Hematol Oncol 2010 3 5 10.1186/1756-8722-3-5 20132536 PMC2827364

[B34] Kim J Guan J Chang I Chen X Han D Wang CY PS-341 and histone deacetylase inhibitor synergistically induce apoptosis in head and neck squamous cell carcinoma cells Mol Cancer Ther 9 7 1977 1984 10.1158/1535-7163.MCT-10-0141 PMC2931416 20571067

[B35] Shen J Huang C Jiang L Gao F Wang Z Zhang Y Bai J Zhou H Chen Q Enhancement of cisplatin induced apoptosis by suberoylanilide hydroxamic acid in human oral squamous cell carcinoma cell lines Biochem Pharmacol 2007 73 12 1901 1909 10.1016/j.bcp.2007.03.009 17445779

[B36] Chung YL Lee MY Pui NN Epigenetic therapy using the histone deacetylase inhibitor for increasing therapeutic gain in oral cancer: prevention of radiation-induced oral mucositis and inhibition of chemical-induced oral carcinogenesis Carcinogenesis 2009 30 8 1387 1397 10.1093/carcin/bgp079 19351790

[B37] Murakami J Asaumi J Kawai N Tsujigiwa H Yanagi Y Nagatsuka H Inoue T Kokeguchi S Kawasaki S Kuroda M Effects of histone deacetylase inhibitor FR901228 on the expression level of telomerase reverse transcriptase in oral cancer Cancer Chemother Pharmacol 2005 56 1 22 28 10.1007/s00280-004-0976-x 15791453

